# Feasibility Study of 3D FACT and IVIM Sequences in the Evaluation of Female Osteoporosis

**DOI:** 10.3390/bioengineering10060710

**Published:** 2023-06-11

**Authors:** Shuo Zhang, Qianrui Guo, Yang Yang, Hongbo Feng, Yan Zhao, Peng Guo, Di Li, Xuemei Du, Qingwei Song

**Affiliations:** 1 Department of Nuclear Medicine, The First Affiliated Hospital of Dalian Medical University, Dalian 116011, China; 2 Beijing United Imaging Research Institute of Intelligent Imaging, Beijing 100094, China; 3 Department of Information Center, The First Affiliated Hospital of Dalian Medical University, Dalian 116011, China; 4 Department of Radiology, The First Affiliated Hospital of Dalian Medical University, Dalian 116011, China

**Keywords:** fat analysis and calculation technique, intravoxel incoherent motion, vertebral bodies, intervertebral discs, bone mineral density

## Abstract

Background: The aim of this study is to search for the predictive value of 3D fat analysis and calculation technique (FACT) and intravoxel incoherent motion (IVIM) parameters in identifying osteoporosis in women. Methods: We enrolled 48 female subjects who underwent 3.0 T MRI, including 3D FACT and IVIM sequences. Bone mineral density (BMD) values and Fracture Risk Assessment (FRAX) scores were obtained. Proton density fat fraction (PDFF) in the bone marrow and the real diffusion (D) value of intervertebral discs were measured on 3D FACT and IVIM images, respectively. Accuracy and bias were assessed by linear regression analysis and Bland–Altman plots. Intraclass correlation coefficients were used to assess the measurements’ reproducibility. Spearman’s rank correlation was applied to explore the correlation. MRI-based parameters were tested for significant differences among the three groups using ANOVA analyses. A receiver operating characteristic (ROC) analysis was performed. Results: The PDFF of the vertebral body showed a negative correlation with BMD (R = −0.393, *p* = 0.005) and a positive correlation with the FRAX score (R = 0.706, *p* < 0.001). The D value of intervertebral discs showed a positive correlation with BMD (R = 0.321, *p* = 0.024) and a negative correlation with the FRAX score (R = −0.334, *p* = 0.019). The area under the curve values from the ROC analysis showed that the 3D FACT and IVIM sequences could accurately differentiate between normal and osteoporosis (AUC = 0.88 using the PDFF; AUC = 0.77 using the D value). The PDFF value demonstrated a sensitivity, specificity, positive predictive value (PPV), and negative predictive value (NPV) of 78.6%, 89.5%, 84.6%, and 85.0%, respectively, in its ability to predict osteoporosis. The D value had a sensitivity, specificity, PPV, and NPV of 63.16%, 92.9%, 65.0%, and 77.8%, respectively, for predicting osteoporosis. Conclusions: The 3D FACT- and IVIM-measured PDFF and D values are promising biomarkers in the assessment of bone quality and fracture risk.

## 1. Introduction

Osteoporosis is a serious health condition that can lead to an increased risk of bone fragility, resulting in disability and mortality [1]. Fractures are more common in women who have undergone menopause, and their incidence varies among different populations [2]. Women are increasingly experiencing vertebral compression fractures due to osteoporosis [3]. Therefore, it is crucial to perform early diagnosis and monitoring to prevent and treat osteoporosis-related fractures in women. 

The World Health Organization (WHO) recommends bone mineral density (BMD) testing accomplished by dual-energy X-ray absorptiometry (DXA) as the gold standard for the diagnosis of osteoporosis [4]. A major disadvantage of DXA-based BMD is the fact that DXA cannot identify changes in the composition of the lumbar spine [5]. In addition, hyperostosis can cause false negative measurements as the increased bone density may mask the actual loss of bone mass, making it difficult to detect osteoporosis or other bone diseases. For instance, in patients with diffuse idiopathic skeletal hyperostosis (DISH), the increased bone density in the spine may interfere with the accurate measurement of BMD using dual-energy X-ray absorptiometry (DXA) and lead to false negative results. Similarly, in patients with Paget’s disease of the bone, excessive bone remodeling may result in false negative BMD results due to increased bone density. Quantitative computed tomography (QCT) may provide more accurate results, but the effective doses to patients from QCT are higher than DXA (0.67~8.8 μSv), ranging from 5 to 3000 μSv depending on the specific protocol and equipment used [6]. Consequently, a noninvasive security technique with excellent accuracy in the diagnosis of osteoporosis is required.

Current functional MRI techniques allow for the noninvasive extraction of tissue composition. Magnetic resonance spectroscopy (MRS) is considered the most precise noninvasive technique for quantifying tissue content. Nevertheless, MRS has several intrinsic constraints, such as being technically difficult, time-consuming, and having a spatial limitation, which, because of the view of MRS, generally covers just a tiny volume of a single voxel. Thus, 3D FACT and IVIM have emerged as widely used techniques that have high repeatability and reproducibility across various readers, field strengths, and MRI platforms [7,8].

The proton density fat fraction (PDFF) of bone marrow is thought to be related to osteoporosis [9] because mesenchymal stem cells differentiate into adipocytes instead of osteoblasts. Margulies [10] suggested that the degeneration of lumbar intervertebral discs (IDD) is also associated with bone mineral density. The reduction of BMD in the lumbar spine results in a decrease in the number of bone trabeculae in the vertebral body, and increased bone fragility affects the nutrient supply to the intervertebral disc and accelerates the degenerative IDD of the intervertebral disc. Given the limited research in these areas, it is highly important to investigate the viability of utilizing these two indicators for the diagnosis of osteoporosis. 

The conventional mono-exponential model and bi-exponential IVIM algorithms are both methods used in diffusion-weighted magnetic resonance imaging (DWI-MRI) to analyze the diffusion of water molecules in tissues. The mono-exponential model assumes that the diffusion of water molecules in tissues follows a single exponential decay curve. It calculates the apparent diffusion coefficient (ADC), which reflects the overall diffusion of water molecules in tissues. However, the mono-exponential model does not take into account the presence of microcirculation in tissues, which can affect the diffusion of water molecules. The bi-exponential IVIM algorithm, on the other hand, takes into account the presence of microcirculation in tissues by modeling the diffusion of water molecules in both the extravascular space and the intravascular space. The bi-exponential IVIM algorithm has the capability to yield the real diffusion, which corresponds to the pure molecular diffusion.

In this work, we simultaneously measured the PDFF of VBs and the D value of IVDs in women. On this basis, we will explore whether the 3D FACT and IVIM sequences can be applied to the opportunistic diagnosis of osteoporosis in women.

## 2. Materials and Methods

### 2.1. Study Population

**Figure 1:**
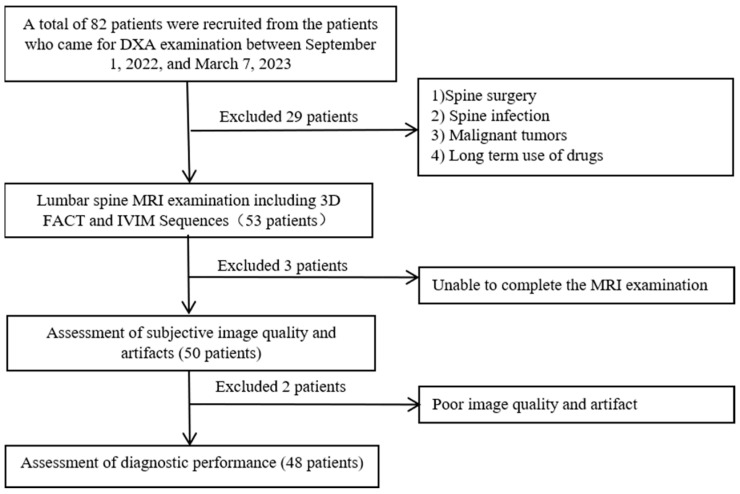
Flow chart of patient enrollment.

Between 1 September 2022, and 7 March 2023, 82 participants were enrolled prospectively and consecutively (Figure 1). MRI and DXA examinations of the lumbar spine for each participant were performed on the same day. A total of 29 patients were excluded for the following reasons: (1) spine surgery; (2) spine infection; (3) malignant tumors; (4) and long-term use of drugs that affect bone metabolism. Three of these patients were disqualified from the study because they were unable to cooperate in completing the MRI examination. Two patients were excluded for poor image quality and artifacts. Finally, a total of 48 participants were collected in the present study. The average age of the cases was 55.9 ± 15.2 years.

### 2.2. BMD Examination

For all the participants, the BMD of the lumbar spine (L1–L4) was evaluated through DXA (Discovery A, Hologic, Marlborough, MA, USA). Before the examination, quality control was performed according to the manufacturer’s standards. During the examination, each subject was in the supine position with both lower limbs elevated, ranging from the lower half of L5 to the upper half of the T12 vertebral body. For patients with severe bone hyperplasia of the lumbar spine, a scan of the hip was performed, and the lowest T value of the two was taken for the diagnosis. The software Hologic version 9.03 was utilized for the analysis of the BMD values. Each test was performed using the same apparatus, which was controlled by a single operator with ten years of experience.

T-scores were used to categorize the patients into three groups according to the World Health Organization’s osteoporosis diagnostic standards [11]: the normal group (T-score ≥ −1.0 SD); the osteopenia group (−2.5 SD < T-score < −1.0 SD); and the osteoporosis group (T-score ≤ −2.5 SD).

### 2.3. FRAX Evaluation

A senior radiologist with twenty years of experience who was qualified by the International Society for Clinical Densitometry (ISCD) assessed the major fracture risk of each participant using the Fracture Risk Assessment Tool (FRAX), which was designed to assess the fracture risk of patients. The models used by FRAX were patient-specific models that incorporated 11 clinical risk factors (i.e., age, sex, weight, height, previous fracture, parental hip fracture, current smoking status, glucocorticoid use, rheumatoid arthritis, secondary OP, and alcohol use of three or more units per day). The FRAX models were built by researching population-based cohorts from Europe, North America, Asia, and Australia.

### 2.4. MR Examination

All participants underwent MRI of the lumbar spine, which was performed with a 3.0T system (uPMR 790, United Imaging Healthcare, Shanghai, China). For pretreatment planning, a routine MR lumbar spine protocol was performed using a 32-channel spine matrix coil.

**Figure 2:**
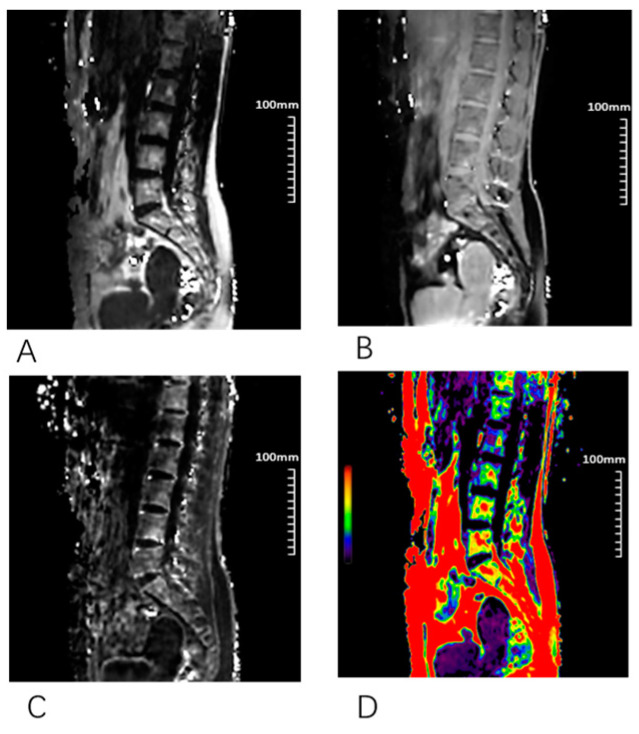
Different sagittal images of 3D fat analysis calculation technique (FACT) sequence. (**A**) Fat; (**B**) water; (**C**) R2*; (**D**) FF.

**Figure 3:**
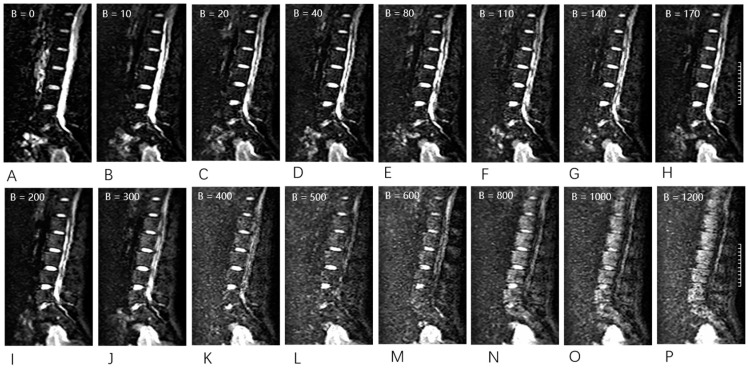
Raw intravoxel incoherent motion (IVIM) sequence data were acquired using 16 different b values (0, 10, 20, 40, 80, 110, 140, 170, 200, 300, 400, 500, 600, 800, 1000, and 1200 s/mm^2^) in sagittal orientation. (**A**–**P**) With an increase in the b value, the signal intensity decreased.

Chemical-shift water–fat images were collected using a 3D gradient-recalled echo FACT sequence with a flyback read gradient to acquire water, fat, fat-fraction, and R2* image series in the sagittal plane of the spine (Figure 2). The FACT sequence employed a low flip angle excitation to reduce T1 bias, several echoes to account for T2 decay, and a calibrated six-peak fat spectrum. The 3D FACT images were acquired with the following parameters: FOV = 400 × 300 mm^2^, matrix = 256 × 192 × 22, pixel size = 1.56 × 1.56 × 8 mm^3^, flip angle = 3°, bandwidth = 900 Hz/pixel, TE = 1.73/3.26/4.79/6.32/7.85/9.38 ms, and TR = 10.94 ms. The single-shot echo planar imaging (SS-EPI) technique was used to acquire IVIM images with 16 b values (0, 10, 20, 40, 80, 110, 140, 170, 200, 300, 400, 600, 800, 1000, and 1200 s/mm^2^) (Figure 3). The acquisition time (min: s) of the 3D FACT and IVIM sequences was 1:01 and 5:30, respectively. 

Nine participants were recruited to study the reproducibility of the 3D FACT and IVIM sequences. Each volunteer was scanned twice. 

### 2.5. Image Analysis

The images were reviewed independently by two radiologists, who did not have access to the clinical radiology reports. Subjective image quality was evaluated on a 4-point scale [12], taking into account the overall quality of the image and the clarity of the VBs and IVDs. This scale was scored as follows: 1 = poor: nondiagnostic and significantly limited evaluation of anatomic structures; 2 = sufficient: adequate for the majority of diagnoses but with somewhat limited evaluation of anatomic structures; 3 = good: adequate for the majority of diagnoses and evaluation of anatomic structures; 4 = excellent: optimal diagnostic utility with precise depiction of the evaluated anatomic structure.

The presence of artifacts was also evaluated on a 4-point scale, taking into account motion, pulsation, and susceptibility, in addition to subjective image noise (1 = severe artifacts; 2 = mild or moderate artifacts affecting anatomic structure evaluation; 3 = mild artifacts not affecting anatomic structure evaluation; 4 = no artifacts).

MRI and DXA evaluations were conducted using a double-blind method. The MRI data were transferred to the United Imaging Workstation (United Imaging Healthcare). The PDFF values of VBs were measured on 3D FACT images. The region of interest (ROI) was placed in the center regions of the VBs on a midline slice of sagittal L1 to L4 while avoiding the effect of vertebral endplate changes to measure the MRI parameters.

The actual apparent diffusion coefficient was generated using the bi-exponential IVIM algorithms. The IVIM theory is based on two individual proton pools that estimate fast and slow diffusion components separately. The following is the signal behavior [13]:

Sb/S0 = (1 − f) × exp (−b × D) + f × exp (−b × D*) (1)

where f is the fractional perfusion associated with microcirculation, D is the real diffusion as indicated by pure molecular diffusion, and D* is the pseudo-diffusion coefficient associated with perfusion. The ROIs of L1/2 to L4/5 IVDs were set while avoiding the upper part of the VBs, the inferior cartilage endplate, and cerebrospinal fluid.

### 2.6. Statistical Analysis

Statistical analysis of the data was performed using SPSS (Statistical Product and Service Solutions) software. *p* < 0.05 was considered statistically significant. Linear regression and Bland–Altman analysis were performed to assess the reproducibility of the 3D FACT and IVIM sequences. Interclass correlation coefficients (ICC) were calculated to assess the interobserver reproducibility of PDFF and D value measurements. MRI-based parameters were tested for significant differences among the three groups using ANOVA analyses. Spearman’s rank correlation was performed to test the correlations. A receiver operating characteristic (ROC) analysis was performed to evaluate the performances of the PDFF and D value In discriminating osteoporosis.

## 3. Results

**Figure 4:**
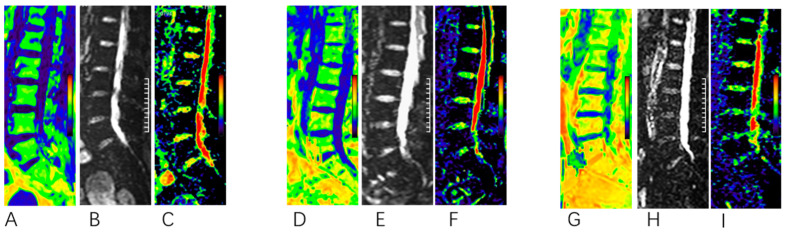
Representative PDFF, IVIM, and D images of 3 subjects ((**A**–**C**), normal bone density; (**D**–**F**), osteopenia; and (**G**–**I**), osteoporosis). (**A**–**C**) A 55-year-old woman with a low PDFF (green regions) and a high D in the lumbar region (red regions). The patient was classified into the normal group according to the DXA results. (**D**–**F**) A 57-year-old woman. The patient was classified into the osteopenia group. (**G**–**I**) A 67-year-old woman with a high PDFF (orange regions) and a low D in the lumbar region (green regions), the patient was classified into the osteoporosis group according to the DXA results.

Data analysis was performed on 48 of the recruited 82 participants, with 34 exclusions due to MR image acquisition and bone disease. Participant characteristics are shown in Table 1. Age, BMI, BMD, PDFF, D, and FRAX scores all described significant differences among the three cohorts, with DXA used as the reference standard. Figure 4 shows representative PDFF maps and D values of the lumbar spines of three subjects; higher PDFF and lower D values were found in more osteoporotic subjects.

### 3.1. Reproducibility

**Figure 5:**
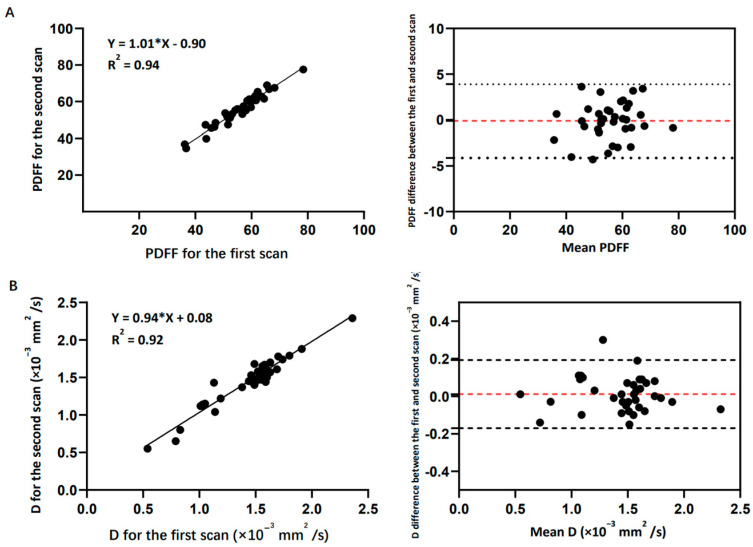
(**A**) Reproducibility results of the 3D FACT sequence: linear regression and Bland−Altman analysis plots of the 3D−FACT−sequence-measured PDFF between the first and the second scans. Mean bias is −0.11 (95% limit of agreement ranges from −4.11 to 3.89). PDFF has no unit. (**B**) Reproducibility results of the IVIM sequence: linear regression and Bland−Altman analysis plots of the IVIM−sequence−measured D value between the first and the second scans. Mean bias is −0.01 (95% limit of agreement ranges from −0.17 to 0.19 × 10^−3^ mm^2^/s).

A total number of 36 VBs and 36 IVDs were analyzed, respectively, to study the reproducibility of the 3D FACT and IVIM sequences. The results of the linear regression and Bland–Altman analyses of the 3D FACT and IVIM sequences for the repeated volunteer scans are shown in Figure 5. Strong correlations and high agreements of the PDFF and D measurements were found between the first and second scans with R^2^ = 0.94 (*p* < 0.001) and R^2^ = 0.92 (*p* < 0.001). The mean bias was −0.11 (the 95% limit of agreement ranged from −4.11 to 3.89) and −0.01 (the 95% limit of agreement ranged from −0.17 to 0.19 × 10^−3^ mm^2^/s). These results demonstrate the excellent reproducibility of the 3D FACT and IVIM sequences.

The interobserver ICCs for the PDFF and D measurements between the two radiologists were 0.97 and 0.95, respectively. The high ICC values demonstrate excellent reproducibility for interobserver measurements. 

### 3.2. Image Quality Assessment

Table 2 presents the scores for subjective image quality and artifacts for the 3D FACT and IVIM sequences for the two readers. The ICCs for the PDFF and D subjective image quality scores between the two readers were 0.99 and 0.95, respectively. The ICCs for the PDFF and D artifacts scores between the two readers were 0.92 and 0.98, respectively.

### 3.3. Correlations and ROC Analysis

The PDFF showed a negative correlation with BMD (R = −0.393, *p* = 0.005) and a positive correlation with the FRAX score (R = 0.706, *p* < 0.001). The D value of intervertebral discs showed a positive correlation with BMD (R = 0.321, *p* = 0.024) and a negative correlation with the FRAX score (R = −0.334, *p* = 0.019) (Table 3).

**Figure 6:**
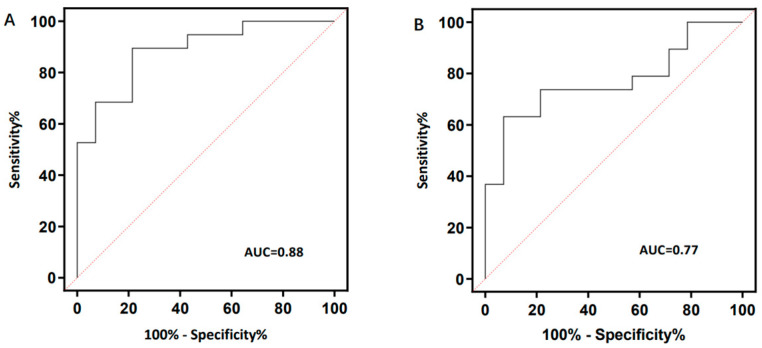
Receiver operating characteristic (ROC) curves and corresponding area under the curve (AUC) values of PDFF (**A**) and D values (**B**) between normal and osteoporosis.

The ROC analysis (Figure 6) shows the ROC curves of the PDFF and D values in differentiating normal individuals from individuals with osteoporosis. The AUC values of PDFF and D were 0.88 (95% CI: 0.776, 0.997) and 0.77 (95% CI: 0.608, 0.933). The PDFF value demonstrated a sensitivity, specificity, positive predictive value (PPV), and negative predictive value (NPV) of 78.6%, 89.5%, 84.6%, and 85.0%, respectively, in its ability to predict osteoporosis. The D value had a sensitivity, specificity, PPV, and NPV of 63.16%, 92.9%, 65.0%, and 77.8%, respectively, for predicting osteoporosis.

## 4. Discussion

In this study, we provided a nondestructive and noninvasive MRI technique for measuring PDFF and D values in the human lumbar spine for assessing bone mineral density, utilizing DXA as a reference standard. For the primary outcome, high AUC values (AUC = 0.88 using the PDFF; AUC = 0.77 using the D value) obtained from ROC analysis showed that the PDFF and D values were capable of discriminating between osteoporosis and normal subjects. The PDFF value had higher sensitivity (78.6%) in predicting osteoporosis, and the D value provided better performance regarding specificity (92.9%). Moreover, the PDFF and D values had correlations with the BMD and FRAX scores. The PDFF of VBs in the osteoporosis group was higher than that of the osteopenia and normal groups; similar findings have been presented in most reports of the PDFF of VBs [14,15]. However, the D values of IVDs were lower in the osteoporosis group than in the osteopenia and normal groups. The lower D values of IVDs in the osteoporosis group resulted largely from lower BMD, and the BMD of lumbar vertebrae was related to IDD [16,17]. This study demonstrates that 3D FACT and IVIM are promising MRI techniques to be used in clinical practice for the assessment of osteoporosis. 

DXA and QCT fall short of providing a comprehensive evaluation of bone strength, which leaves many high-risk patients without the opportunity for early intervention. Bone quality encompasses changes in bone microstructure and molecular levels, with the dynamic alteration of marrow content and IVDs closely tied to bone remodeling capacity. Fatty degeneration of bone marrow can impede osteoblast generation and lead to thin and residual bone trabeculation. Our findings align with a study of 400 healthy individuals that revealed a negative correlation between PDFF values and BMD, even after adjusting for variations in bone marrow adipose composition and age [18].

In this study, the D value was used to reflect the degree of intervertebral disc degeneration, and the study showed that lumbar disc degeneration had a positive correlation with BMD. The same results showed up in the studies by Pan [19] and Margulies [10]. The possible causes were analyzed as follows: (1) lower BMD affected bone microcirculation, resulting in insufficient blood supply of the intervertebral disc and accelerating its degeneration, which can also cause a decrease in BMD; (2) lower BMD can cause microfractures of the subchondral bone plate of the lumbar spine, leading to the destruction of microvessels, affecting the vertebral body and intervertebral disc, and promoting the degeneration of intervertebral discs at the same time; (3) a lack of estrogen promotes the occurrence and development of osteoporosis and lumbar intervertebral disc degeneration. After menopause, estrogen decreases, seroprotection production decreases, and its inhibitory effect on osteoclasts decreases, leading to osteoporosis and intervertebral disc degeneration. However, some researchers [20,21] believe that BMD is positively correlated with the degree of lumbar disc degeneration. The higher the BMD, the more severe the disc degeneration. Further studies should be carried out in combination with the 3D finite element model of the lumbar spine in osteoporosis patients and normal subjects and its biomechanical properties.

In the past decade, the important role of PDFF of VBs in bone loss prevention was proposed by a series of studies [22,23,24]. According to Schwartz et al.’s research [25], high levels of PDFF are linked to lower BMD and an increased risk of fractures. Patsch et al.’s study [26] also indicates that PDFF composition is associated with fragility fractures, and spinal bone marrow fat could be a novel tool for assessing fracture risk independent of BMD. This negative correlation between PDFF and BMD may be due to a reduction in bone-forming cells as the amount of fat in the bone marrow increases, leading to a decrease in bone density. Additionally, hormones produced by fat cells can impact bone metabolism, further affecting bone density. Bredella et al.’s study [27] suggests that the increase in adipose tissue in obese individuals leads to a decrease in bone load, which affects bone density formation and maintenance, resulting in a negative correlation between PDFF values and bone density. This implies that reducing bone marrow fat could be a potential therapeutic target for improving bone health. Nonetheless, more research is necessary to comprehend the intricate relationship between bone marrow fat and bone density. 

Due to the diverse distribution of bone marrow in most places, high-resolution PDFF mapping is very beneficial. When evaluating the PDFF of bone marrow using water–fat imaging, various confounding issues have to be addressed, including the existence of several peaks in the fat spectrum, T1 bias, and T2* decay effects [28,29]. In the present study, PDFF images were acquired utilizing the 3D FACT sequence to increase the precision of MR parameter measurement. In this work, a variable projection was used to overcome the constraints of classic region-growing approaches and multiresolution techniques for robust fat–water separation [30,31].

The water content of the annulus fibrosus and nucleus pulposus tissues reaches 70–80%. IDD mainly manifests as a decrease in water content. Several previous studies using DWI have reflected changes in the random mobility of water molecules in tissues [32,33], which suggested that DWI may be utilized as a noninvasive tool for assessing degenerative changes in IVDs. Compared to the conventional mono-exponential model, the bi-exponential IVIM algorithm provides more information about tissue microcirculation. However, the bi-exponential model is more complex and requires longer acquisition times, which can limit its clinical use. IVIM became clinically accessible following its connection with echo-planar imaging (EPI) since signals captured at numerous and higher b values could be obtained without motion artifacts [34], enabling the first clinical validation of IVIM perfusion MRI in a set of patients with liver lesions [35]. The ADC value indicates the distribution of diffusion-driven displacements in the conventional mono-exponential model. Instead of traditional ADC, the diffusion coefficient D and perfusion-related coefficients were evaluated independently in this study, allowing for the more precise evaluations of IVDs.

Our findings show that the PDFF and D values may provide additional information on bone fragility. The result suggests that the PDFF and D values had a negative and positive correlation, respectively, with the FRAX score. This method would therefore support physicians’ decisions regarding bone stability and the individual treatment selection for each patient.

This study has several limitations. Firstly, the sample size assessed in this study was fairly small. Therefore, future studies with larger study cohorts are needed to confirm our findings. Subsequently, subgroup analysis was not conducted, so the results may be biased. We will assess the influence of age factors, medication, comorbidities and further medical history in future studies to address this limitation. Finally, the MRI scanning parameters applied in this study may not be optimal, and the manual mapping of ROI could also affect the parameter quantification performances. We will try to standardize the MRI scanning parameters and the ROI mapping in the future.

## 5. Conclusions

In summary, the PDFF and D values were shown to be highly accurate and reproducible in MRI. The 3D FACT and IVIM sequences are feasible in the evaluation of female osteoporosis, and the 3D-FACT- and IVIM-derived parameters have the potential to be used as reliable predictors to detect fracture risk in women.

## Figures and Tables

**Table 1:** Comparisons of different parameters in the normal, osteopenia, and osteoporosis groups.

	All Subjects (*n* = 48)	Normal (*n* = 14)	Osteopenia (*n* = 15)	Osteoporosis (*n* = 19)	*p*
Ages (years)	55.9 ± 15.2	46.4 ± 12.7	58 ± 16.2	66.5 ± 8.3	<0.05 *
BMI (kg/m^2^)	23.7 ± 3.5	23.8 ± 3	23.3 ± 3.4	24 ± 4.5	0.860
BMD (mg/cm^2^)	918 ± 148	1062 ± 69.1	877 ± 62.5	766.8 ± 98.1	<0.05 *
PDFF (%)	50.4 ± 12.5	41.7 ± 11.1	55.3 ± 12.6	56.8 ± 6.7	<0.05 *
D (×10^−3^ mm^2^/s)	1.4 ± 0.3	1.6 ± 0.2	1.5 ± 0.58	1.2 ± 0.2	<0.05 *
FRAX score (%)	3.6 ± 2.8	2.6 ± 2.2	3.8 ± 2.6	5.4 ± 2.8	<0.05 *

All values are expressed as mean ± SD. T score, PDFF, and FRAX score have no unit. * *p* < 0.05 was considered significant.

**Table 2:** Subjective Image Quality and the Artifacts of the 3D FACT and IVIM MRI Techniques.

Subjective Image Quality	3D FACT (Reader1/2)	IVIM (Reader1/2)
Grade 1	0/0	1/1
Grade 2	2/2	0/0
Grade 3	45/39	90/105
Grade 4	128/136	68/48
Artifacts		
Grade 1	0/0	1/1
Grade 2	2/2	0/0
Grade 3	93/66	108/120
Grade 4	64/100	44/28

Data are the sum of scores for readers 1 and 2, respectively.

**Table 3:** The correlations among different parameters.

Parameters	BMD	FRAX
PDFF		
R	−0.393 **	0.706 **
*p*	0.005	<0.001
D		
R	0.321 *	−0.334 *
*p*	0.024	0.019

* Correlation was significant at the 0.05 level (two-tailed). ** Correlation significant at 0.01 level (two-tailed).

## Data Availability

The datasets generated or analyzed during the study are available from the corresponding author upon reasonable request.
